# The unexpected silent manifestation of myocardial infarctions in ischemic heart failure patients: Insights from a case-control study^☆^

**DOI:** 10.1016/j.clinsp.2024.100480

**Published:** 2024-08-30

**Authors:** Gabriel Cordeiro Polo Mendes, Paulo Cury Rezende, Arthur Cicupira Rodrigues de Assis, Vitor Coutinho Andrade, Thiago Luis Scudeler, Marcela Francisca da Silva, Mauricio Rigodanzo Mocha, Whady Hueb, Jose Antonio Franchini Ramires, Roberto Kalil Filho

**Affiliations:** Instituto do Coração (InCor), Hospital das Clínicas, Faculdade de Medicina, Universidade de São Paulo, São Paulo, SP, Brazil

**Keywords:** Myocardial infarction, Case control study, Type 2 diabetes mellitus, Diabetic neuropathy

## Abstract

**Background:**

Recent studies show Silent Myocardial Infarction (SMI) as a quite frequent event. However, regarding severe tertiary care patients that frequently present consequences of Coronary Artery Disease (CAD) and Left Ventricular Dysfunction (LVD), the occurrence of this manifestation is unexpected and its associated factors aren't clear in the literature.

**Aim:**

To compare clinical, laboratorial, ventricular and angiographic factors between silent and classical presentation of MI in patients with CAD and LVD.

**Methods:**

Patients with multivessel CAD with over 70 % obstructive lesions and LVD with EF less than 35 % were evaluated for MASS VI trial and later included in the present study. The ventricular function and coronary assessment were measured by echocardiography and SYNTAX score, respectively. The population was stratified in a SMI group and Clinically Manifested Myocardial Infarction (CMMI) group based on MI presentation for a comparison of medical parameters.

**Results:**

From 132 patients, 47 (35.6 %) were classified as SMI and 85 (64.4 %) as CMMI. No differences were observed between groups regarding age, sex, diabetes mellitus, SYNTAX score, or collateral circulation. Higher proportion of NYHA II classification, inferior wall MI and lower creatinine clearance were found in SMI group. After multivariate analysis, peripheral diabetic neuropathy (OR = 4.6 [1.1‒12.7] p = 0.032) and inferior wall MI (OR = 4.1 [1.5‒11.4] p = 0.007) were significantly associated with SMI.

**Conclusion:**

Peripheral diabetic neuropathy and inferior wall MI were associated with SMI presentation. Overall, associated factors tend to be similar comparing SMI and CMMI, but in the specific population of diabetic patients with chronic neuropathy a special care should be taken.

## Highlights

• Silent and Clinically Manifested Myocardial infarctions have similar associated factors.
• Age, sex, hypertension, and diabetes are similarly distributed in both groups.
• Silent myocardial infarction is associated with peripheral diabetic neuropathy.
• Silent myocardial infarction is associated with inferior wall topography.

## Graphical abstract

**Figure:**
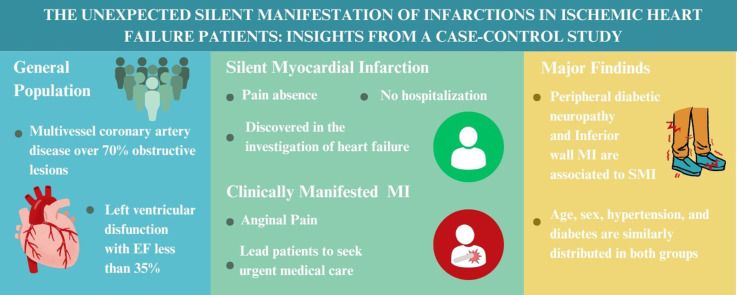

## Introduction

The classical presentation of Myocardial Infarction (MI) is characterized by anginal features such as retrosternal, precordial, or epigastric pain, often described as a pressure or burning sensation with radiation, sweating, and worsening upon exertion. However, a significant proportion of myocardial infarction patients present atypical symptoms or even without any symptomatic manifestation of an acute coronary syndrome. In relevant cohort studies, a high prevalence of Silent Myocardial Infarction (SMI) is becoming more noticeable, accounting for 20 % ^1^ to 44 % ^2^ of all MI. In these patients, diagnosis is made through routine clinical tests such as electrocardiography and echocardiography, or during the investigation of effort-related dyspnea in patients who develop heart failure, one of the possible complications following MI.

In the literature there is controversy and little information on the associated factors to this specific manifestation of infarction. The incidence and prevalence of SMI in some populations such as patients with hypertension and diabetes have been studied in a recent meta-analysis,^3^ but ventricular, laboratorial and angiographic factors are mostly undescribed and there is no study that accounts for a control group comparison.

Thus, there is a lack of well-founded understanding on descriptive and related aspects of SMI, especially in terms of study designs that can clarify these uncertainties. Therefore, we propose a case control study to compare the incidence of several factors in a SMI population compared to a Clinically Manifested Myocardial Infarction (CMMI) group.

## Objectives

To compare clinical, laboratorial, ventricular and angiographic factors associated with silent myocardial infarction to those present in patients with clinically manifested myocardial infarction in patients with coronary artery disease and left ventricular dysfunction.

## Methods

### Study design

The present study is a subanalysis of the “Hypotheses, rationale, design, and methods for prognostic evaluation of a randomized comparison between patients with coronary artery disease associated with ischemic cardiomyopathy who undergo Medical or Surgical treatment: MASS-VI (HF)”.^4^ The MASS-VI trial aims to prospectively compare two groups of patients over two treatment options: surgical revascularization or medical therapy. Inclusion criteria are multivessel coronary artery disease with over 70 % obstructive lesions and left ventricular dysfunction with ejection fraction measurements less than 35 %. In addition, myocardial ischemia assessed as angina symptoms or documented evidence of ischemia by stress-testing methods (treadmill stress tests, myocardial scintigraphy, stress-echocardiography, and cardiac magnetic resonance) are needed for inclusion. The events considered for analysis are all-cause mortality, nonfatal infarction, unstable angina requiring additional revascularization, and stroke. The events are being analyzed according to the intention-to-treat principle.

**Fig. 1:**
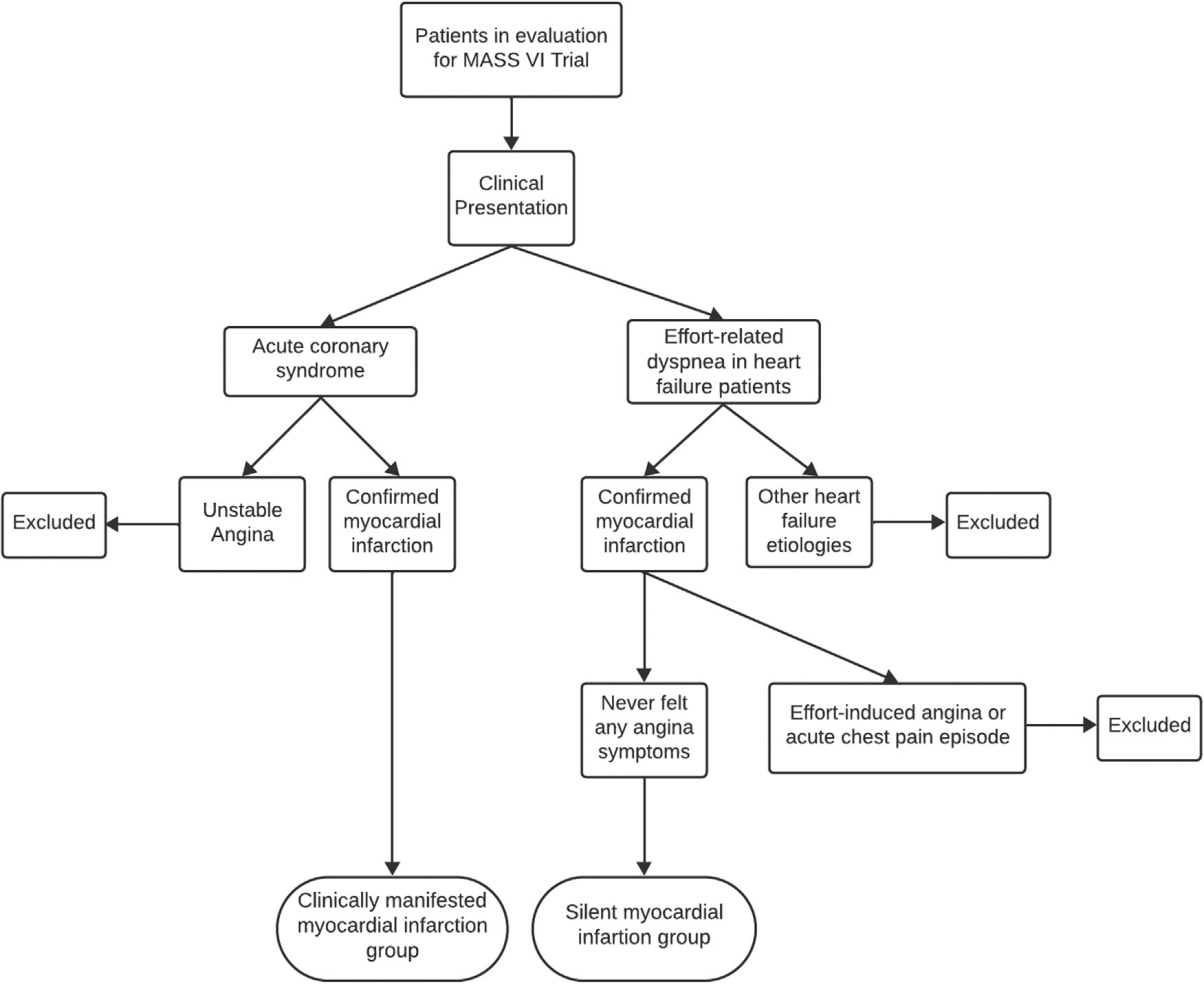
Patient selection flowchart.

The current analysis is a case-control study that assessed patients who were evaluated for inclusion in the MASS-VI trial (“Medicine, Angioplasty, or Surgery Study”), from “Instituto do Coração do Hospital das Clínicas da Faculdade de Medicina da Universidade de São Paulo”. From these data, patients were stratified in two groups according to the presence or not of symptoms of myocardial infarction, one included patient with clinically manifested myocardial infarction and the other patients with silent myocardial infarction (Fig. 1).

In terms of the standardization of clinically manifested myocardial infarction diagnosis, patients exhibited a classical history of acute anginal symptoms, that led the patients to seek an urgent medical care, and where myocardial infarction was confirmed through electrocardiography and cardiac injury biomarkers measurements. With this clinical presentation, they were referred to InCor-HCFMUSP. These patients were evaluated for inclusion in the MASS VI trial because they had multivessel CAD and reduced ejection fraction. Regarding the standardization of silent myocardial infarction diagnosis, most patients with heart failure during the investigation of effort-related dyspnea found electrically inactive areas by electrocardiography, segmental hypokinesia or akinesia by echocardiography or segmental hypocaptation by myocardial scintigraphy. Fewer proportion of SMI were detected through routine electrocardiography in primary care, leading to patient referrals to InCor-HCFMUSP. Notably, these patients did not report any history of anginal symptoms. Further investigation, including cineangiocoronariography, transthoracic echocardiography, myocardial scintigraphy, and cardiac magnetic resonance imaging, confirmed myocardial infarction in both patient groups. Patients with unclear clinical history or who presented effort-induced angina, even without an acute manifestation, or with atypical symptoms went through a reanalysis with at least one new researcher review, then were excluded from inclusion. Figure 1 shows the flowchart of the inclusion of patients in the study.

### Collected data

Four categories of data were collected by medical records or during outpatient visit from the two groups of patients: clinical, laboratorial, ventricular and angiographic parameters.

Clinical parameters:

• Age: Assessed at the moment of the myocardial infarction event for CMMI group and the age of the beginning of effort-related dyspnea for SMI group.
• Gender.
• Dyslipidemia: Reported by medical records and whenever patients were on statins and had LDL-cholesterol levels > 130 mg/dL.
• Systemic Arterial Hypertension (SAH): Reported by medical records and whenever patients were on anti-hypertensive therapy
• Type 2 Diabetes Mellitus (T2DM): Reported by medical records or whenever patients were on oral hypoglycemic drugs and/or insulin.
• Tobacco smoker: Reported by medical records or during outpatient visit.
• Peripheral diabetic neuropathy: Reported by medical records; associated or not with the use of Gabapentin or Pregabalin; or by the use of the modified Neuropathy Symptom Score (NSS) ^5^ translated to portuguese ^6^ by phone calls to all diabetic patients without neuropathic symptoms mention on medical record. Patients were asked about their experience of pain or discomfort in the legs: if the patient described burning, numbness or tingling a score of 2 was assigned; fatigue, cramping or aching scored 1. The presence of symptoms in the feet was assigned a score of 2, the calves 1 and elsewhere a score of 0. Nocturnal exacerbation of symptoms scored 2, 1 for both day and night, and 0 for daytime alone. A score of 1 was added if the symptoms had ever woken the patient from sleep. The patients were asked if any maneuver could reduce the symptoms: walking was assigned a score of 2, standing was 1 and sitting or lying down was 0. The maximum symptom score was 9. For the present study, only patients with moderate or severe symptoms in the score (at least 5 on scale) were considered, thus all of them experienced at least burning, numbness or tingling; presence of symptoms in the feet; or nocturnal exacerbation of symptoms.
• New York Heart Association functional classification (NYHA class): Information assessed by medical record in the earlier record after the infarction event for CMMI group and in the first record for SMI group
• Canadian Cardiovascular Society (CCS) angina class: Information assessed by medical record in the earlier record after the infarction event for CMMI group and in the first record for SMI group
• Chronic kidney insufficiency: Patients with creatinine clearance < 60 mL/min/1.73 m^2^.

Laboratorial parameters:

• Serum creatinine level: The first measurement after medical discharge from myocardial infarction event for CMMI group and the earlier measurement at InCor/HCFMUSP for SMI group
• Creatinine clearance: Calculated by CKD-EPI creatinine equation for the estimation of glomerular filtration rate
• Glycosylated Hemoglobin (HbA1c): The first measurement after medical discharge from myocardial infarction event for CMMI group and the earlier measurement at InCor/HCFMUSP for SMI group
• Low-Density Lipoprotein (LDL): The first measurement after medical discharge from myocardial infarction event for CMMI group and the earlier measurement at InCor/HCFMUSP for SMI group

Ventricular parameters:

• Ejection Fraction (EF): Measured by transthoracic echocardiography after medical discharge from myocardial infarction event for CMMI group and the earlier measurement at InCor/HCFMUSP for SMI group.
• Diffuse hypokinesis: Information assessed in transthoracic echocardiography report.
• Infarction Location ‒ anterior, inferior and/or lateral wall: Information assessed through a combined interpretation of ECG, echocardiogram and scintigraphy.
  ○ ECG criteria: Characterized by the presence of pathological Q waves (abnormally wide [> 0.2 second] and abnormally deep [> 5 mm]) in two contiguous leads ‒ following the III SBC Guidelines.^7^

Anterior Wall:

• Anteroseptal wall ‒ V1, V2, and V3 leads.
• Anterior wall ‒ V1, V2, V3, and V4 leads.
• Localized anterior wall ‒ V3, V4 or V3-V5 leads.
• Anterolateral wall ‒ V4 to V5, V6, D1, and aVL leads.
• Extensive anterior wall ‒ V1 to V6, D1, and Avl.

Lateral Wall:

• Low lateral wall ‒ V5 and V6 leads.
• High lateral wall ‒ D1 and aVL

Inferior Wall: inferior wall - D2, D3, and aVF

• Observation: Anterolateral wall, and extensive anterior wall were considered both anterior and lateral infarctions.
  ○ Echocardiographic criteria: segmental hypokinesis or akinesis in anterior, lateral or/and inferior ventricular wall by transthoracic echocardiography report. Septal dysfunction was considered as anterior wall.
  ○ Myocardial scintigraphy criteria: segmental hypocaptation in anterior, lateral or/and inferior ventricular wall by myocardial scintigraphy. Septal hypocaption was considered as anterior wall.

Angiographic parameters:

• SYNTAX score I: Assessed with the use of the SYNTAX calculator.^8^ Difficult cases were also analyzed by a second experienced researcher.
• Severe involvement of the anterior descending artery: Obstruction of over 70 % in proximal segment of anterior descending artery (before the emergence of the first septal artery).
• Diffuse coronary lesions: At least 75 % of the length of any segment proximal to the lesion, at the site of the lesion or distal to the lesion with a diameter of < 2 mm due to atherosclerosis
• Presence of collateral circulation: Assessed by coronary angiography.

### Statistical analysis

The description of the results for quantitative variables used means and standard deviations or medians and interquartile ranges, depending on the distribution of the variables. Categorical variables are presented through absolute and relative frequencies. Quantitative variables were analyzed for normality using the Shapiro-Wilk test. Those with a symmetric distribution were compared between groups using the Student's *t*-test, and those with an asymmetric distribution were compared using the Mann-Whitney test. The association of clinical, laboratory, angiographic, and ventricular factors with the occurrence of silent myocardial infarction were performed using a multivariate logistic regression model, where the variables included were those that have shown statistical significance (p < 0.20) and those that demonstrate biological plausibility. All analyses were performed in JASP 0.17.3 version. All tests were 2-sided, and a p < 0.05 was considered statistically significant.

## Results

A total of 132 patients were included in the study. Of these, 47 (35.6 %) were included in the SMI group and 85 (64.4 %) in the CMMI group. Clinical, laboratorial, ventricular and angiographic data are depicted in Table 1. Of note, statistical significant differences were found between the two groups in terms of dyslipidemia, NYHA classification, CCS angina class, diffuse hypokinesis, anterior wall MI, inferior wall MI and serum creatinine levels. Briefly, NYHA classification I was more frequent in CMMI group and NYHA II was significantly greater in SMI group. Regarding CCS angina class, a higher proportion of effort-induced angina after myocardial infarction was observed in CMMI group. In terms of anatomical findings, the proportion of inferior wall MI was greater in SMI group and anterior wall MI was greater in CMMI group.

**Table 1:** Baseline characteristics of study population, stratified by MI presentation.

Parameters	Clinically manifested myocardial infarction	Silent myocardial infarction	p-value
*Clinical Parameters*			
Age	59.4 (±8.2)	62.2 (±8.2)	0.60
Male sex	58 (68.2 %)	32 (68.1 %)	0.986
Dyslipidemia	67 (78.8 %)	29 (61.7 %)	0.034
Systemic Arterial Hypertension	72 (84.7 %)	41 (87.2 %)	0.692
Type 2 diabetes mellitus	52 (61.2 %)	30 (63.8 %)	0.763
Peripheral diabetic neuropathy	8 (9.4 %)	10 (21.3 %)	0.081
Smoking index	15 (0‒40)	30 (0‒40)	0.211
Chronic kidney insufficiency	19 (22.3 %)	16 (34.0 %)	0.125
Tobacco smoker	30 (35.3 %)	16 (34.0 %)	
Former smoker	27 (31.8 %)	19 (40.4 %)	0.398
Non smoker	27 (31.8 %)	10 (21.3 %)	
NYHA I	40 (47.6 %)	12 (25.5 %)	
NYHA II	31 (36.5 %)	28 (59.6 %)	0.009
NYHA III	14 (16.5 %)	5 (10.6 %)	
NYHA IV	0 (0 %)	2 (4.2 %)	
CCS 0	44 (51.8 %)	42 (89.4 %)	
CCS I	21 (24.7 %)	2 (4.3 %)	
CCS II	13 (15.3 %)	3 (6.4 %)	<0.001
CCS III	6 (7.1 %)	0 (0 %)	
CCS IV	1 (1.2 %)	0 (0 %)	
*Laboratorial parameters*			
Serum creatinine level	1.1 (0.9‒1.2)	1.1 (1‒1.4)	0.245
Creatinine clearance	71.1 (±18.2)	66.5 (±17.9)	0.048
HbA1C	6.5 (5.9‒7.9)	6.6 (5.9‒9)	0.823
LDL	92 (74‒125)	80.5 (68.5‒106)	0.156
*Ventricular parameters*			
Ejection Fraction	35.2 (±6.0)	35.1 (±5.8)	0.867
Diffuse hypokinesis	7 (8.2 %)	12 (25.5 %)	0.005
Anterior MI	57 (67.1 %)	18 (38.3 %)	0.003
Inferior MI	35 (41.2 %)	36 (76.6 %)	<0.001
Lateral MI	8 (9.4 %)	7 (14.9 %)	0.331
*Angiographic parameters*			
SYNTAX score	25.1 (±7.2)	25.9 (±8.4)	0.594
Left anterior descending artery over 70 % obstruction	50 (58.8 %)	28 (59.6 %)	0.769
Diffuse coronary lesions	18 (21.2 %)	10 (21.3 %)	0.965
Collateral circulation	53 (62.3 %)	31 (66.0 %)	0.701
Uniarterial CAD	5 (5.9 %)	1 (2.1 %)	
Biarterial CAD	10 (11.8 %)	8 (17.0 %)	0.446
Triarterial CAD	67 (78.8 %)	35 (74.5 %)	

**Fig. 2:**
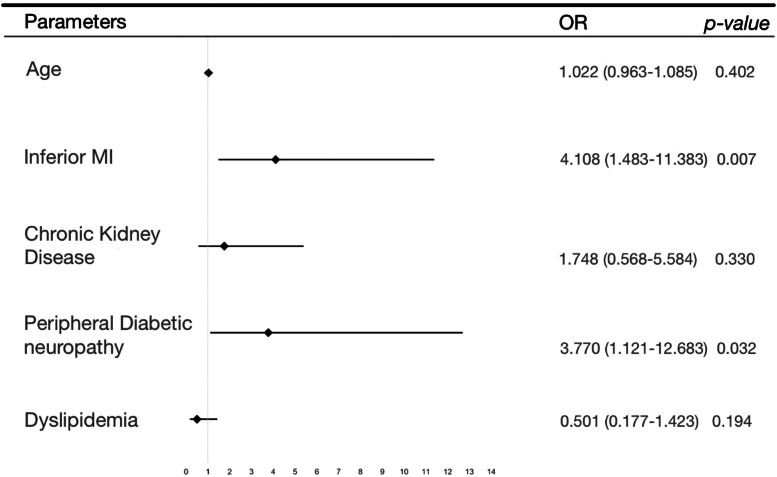
Results of multivariate analysis.

The results of the multivariate analysis are shown in Table 2 and represented in a Forrest plot in Figure 2. Of note, it shows a significant association of peripheral diabetic neuropathy (OR = 4.6 [1.1‒12.7]; p = 0.032) and inferior wall MI (OR = 4.1 [1.5‒11.4]; p = 0.007) with silent myocardial infarction presentation.

**Table 2:** Multivariate analysis.

	Wald Test				95 % Confidence Interval	
	Estimate	Standard error	Odds Ratio	p-value	Lower bound	Upper bound
(Intercept)	-2.593	1.937	0.075	0.181	0.002	3.330
Age	0.22	0.030	1.022	0.472	0.963	1.085
Inferior MI	1.413	0.520	4.108	0.007	1.483	11.383
Chronic kidney insufficiency	0.559	0.574	1.748	0.330	0.568	5.384
Peripheral diabetic neuropathy	1.327	0.619	3.770	0.032	1.121	12.683
Dislipidemia	-0.690	0.532	0.501	0.194	0.177	1.423

## Discussion

The present study evaluated CAD patients who were referred to a cardiology tertiary center after an episode of myocardial infarction and assessed whether they had or not symptoms of an acute coronary syndrome. We found a significant proportion of patients with silent myocardial infarction and compared their clinical, ventricular and laboratorial parameters with patients with typical presentation of myocardial infarction.

The major finding in this study was that peripheral diabetic neuropathy is associated with silent myocardial presentation. Even after the multivariate analysis adjusted for important covariates, we found a significant association. This evidence supports that the overall damage in the nervous system caused by chronic hyperglycemia levels also interferes with the heart cardiac pain perception. Counterintuitively, the presence of diabetes was not statistically significant when the groups were compared - evidence already seen in other studies ^9^^,^^10^ ‒ contributing to the hypothesis that only later stages of this disease with severe damage can modify MI presentation. These findings are not consistent in the literature. Silent myocardial ischemia was found to be related to neuropathic dysfunction in at least two studies,^11^^,^^12^ but its results and biological implications were criticized later.^13^ The FIELD study,^14^ a large cohort that aimed to calculate the incidence and factors associated with SMI, and that evaluated MI based only on ECG findings, the authors did not find neuropathy to be a predictor, but in their methods they did not count non Q-waves infarction, which play a significant role in these patients. In our analysis it was not infrequent to observe patients with normal ECGs but with altered myocardial scintigraphy and/or transthoracic echocardiogram with coronary anatomical findings that were supported by angiography. This limitation of the FIELD study methodology might explain its differences with our study. Regarding gender, we found no difference between the groups. Although our sample size is not very high, the frequency of male sex is quite similar (68.1 %‒SMI and 68.2 %‒CMMI). This result shows that although male sex is a risk factor for acute coronary events, it seems not to influence its presentation. The same reasoning can be applied to other clinical aspects such as hypertension, tabagism and others. This supports in part some findings of the FIELD study ^14^ that showed no statistical relevance for any clinical predictor for SMI.

A relevant ventricular finding was that myocardial infarction in the inferior wall was associated with silent presentation and this was observed even after multivariate adjustment. This finding may be related to anatomical aspects of inferior myocardial wall such as the smaller amount of muscle mass, the innervation or even the right coronary vascularization. It is important to emphasize that a survival bias may exist in this interpretation. SMI of the anterior wall may have higher chances of evolving with sudden death and/or hospital severe complications, and this may reduce the chances of such patients being referred to our cardiology center. Thus, this might reduce the proportion of anterior wall SMI found in this study, and magnify the proportion of inferior wall SMI patients.

### Limitations

Overall, it has a small sample size. Thus, a lot of the statistical power and confidence is subtracted from our analysis. Another aspect is that despite our effort to precisely revise all patients' medical records to define study groups and exclude doubtful cases, patient history may still be subjected to recall bias. On the other hand, although this was a retrospective study, most patients are followed by the MASS-VI trial, and could be reassessed regarding their data. Concerning peripheral diabetic neuropathy diagnosis, we used a symptom score that is frequently applied and has a good accuracy in the literature.^5^ Although a combination with a neurological physical exam would add value, the use of it alone or independently of the physical exam has been validated,^15^ and has been utilized by other studies.^16^, ^17^, ^18^

## Conclusion

In this study, peripheral diabetic neuropathy and inferior wall were associated with silent myocardial infarction presentation. Overall, associated factors tend to be very similar comparing SMI and CMMI, but in the specific population of diabetic patients with chronic neuropathy a special care should be taken.

## Availability of data and materials

The datasets used and analyzed during the current study are available from the corresponding author on reasonable request.

## Disclosure statement

None of the authors of this study has a financial or any other relation that would pose a conflict of interest.

## Declaration of competing interest

The authors declare no conflicts of interest.
